# Supplementary material for: Assessment of Various Food Proteins as Structural Materials for Delivery of Hydrophobic Polyphenols Using a Novel Co-Precipitation Method
Source: Molecules. 2023 Apr 19;28(8):3573. doi: 10.3390/molecules28083573 (PMC10147046; doi:10.3390/molecules28083573)
Supplement: Supplementary file 1 [file molecules-28-03573-s001.zip › molecules-2237933-supplementary.pdf]

Supplementary Materials

Assessment of various food proteins as structural materials for delivery of hydrophobic polyphenols using a novel co-precipitation method

Ali Rashidinejad[a*], Matthijs Nieuwkoop[a©], Harjinder Singh[a] and Geoffrey B. Jameson[a,b]

[a]Riddet Institute, Massey University, Private Bag 11 222, Palmerston North, New Zealand.
[b]School of Natural Sciences, Massey University, Palmerston North 4442, New Zealand.
[*]Corresponding author: A.Rashidinejad@massey.ac.nz.
[©]Current Address: Jan Zandbergen Group, Veenendaal, The Netherlands.

Polyphenol | **Polyphenol precipitate** | **Polyphenol-trehalose precipitate**

Rutin

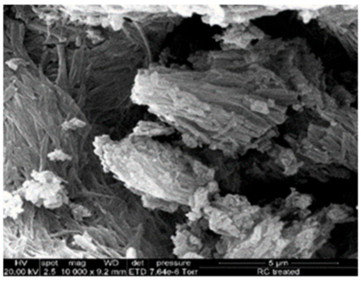
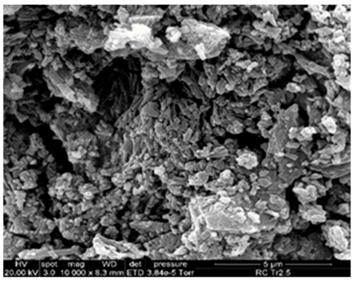

Naringenin

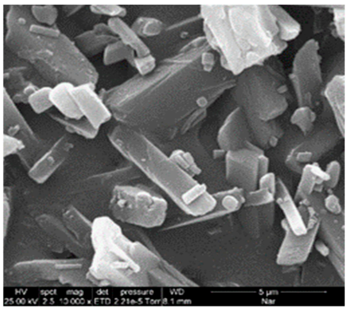
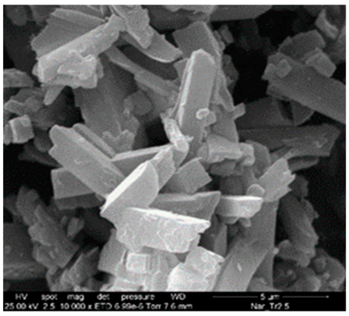

Hesperidin

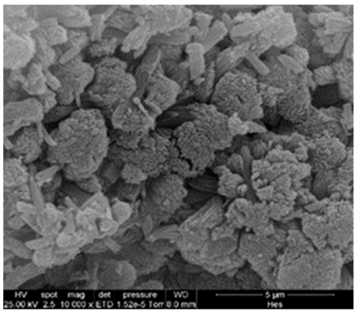
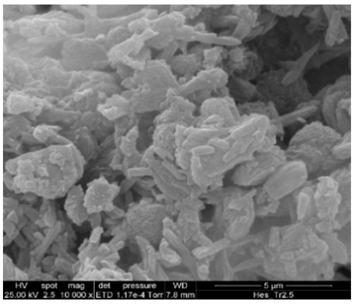

Curcumin

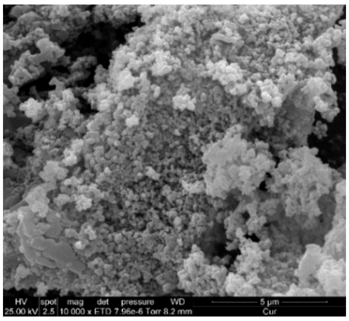
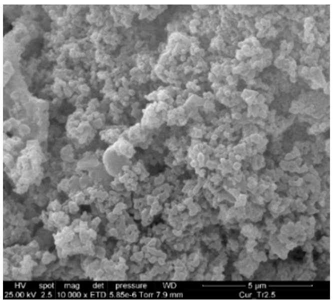

Catechin

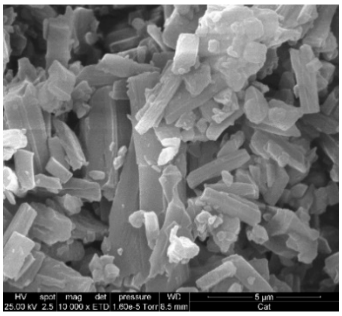
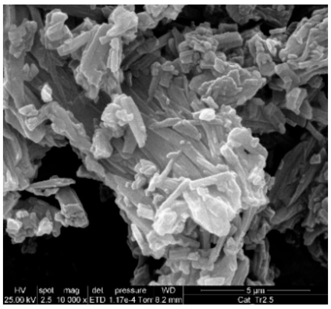

Figure S1. Scanning electron micrographs of the powders of treated polyphenols (precipitates) in the absence and presence of trehalose. The scale bars can be found at the bottom of each micrograph.

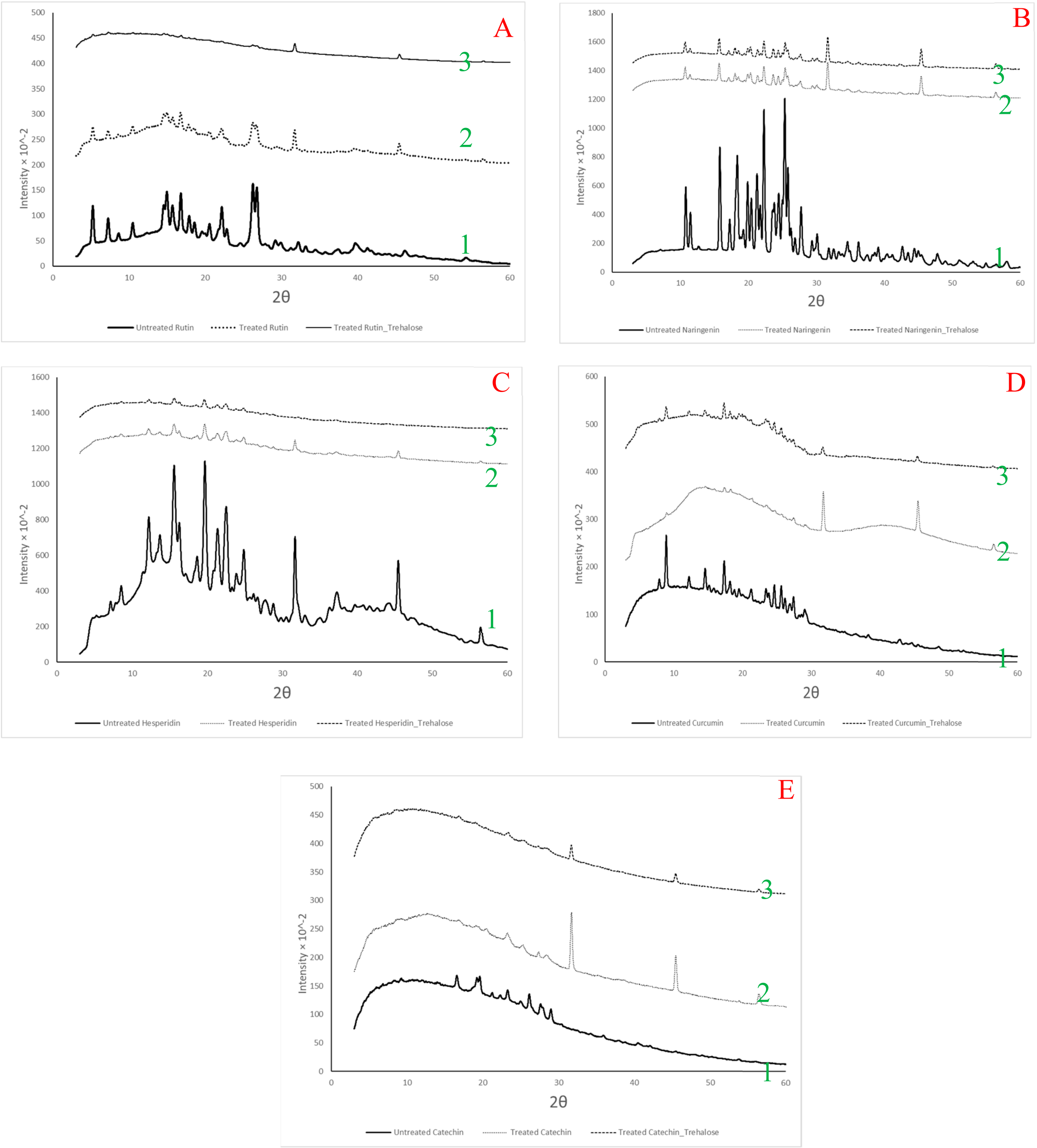

Figure S2. X-ray diffraction patterns of the powders of treated polyphenols (precipitates) in the absence and presence of trehalose. A; rutin, B; naringenin, C; hesperidin, D; curcumin, E: catechin. Legends from bottom to top: 1; untreated flavonoid, 2; treated in the presence of neither protein nor trehalose, 3; treated in the presence of trehalose.

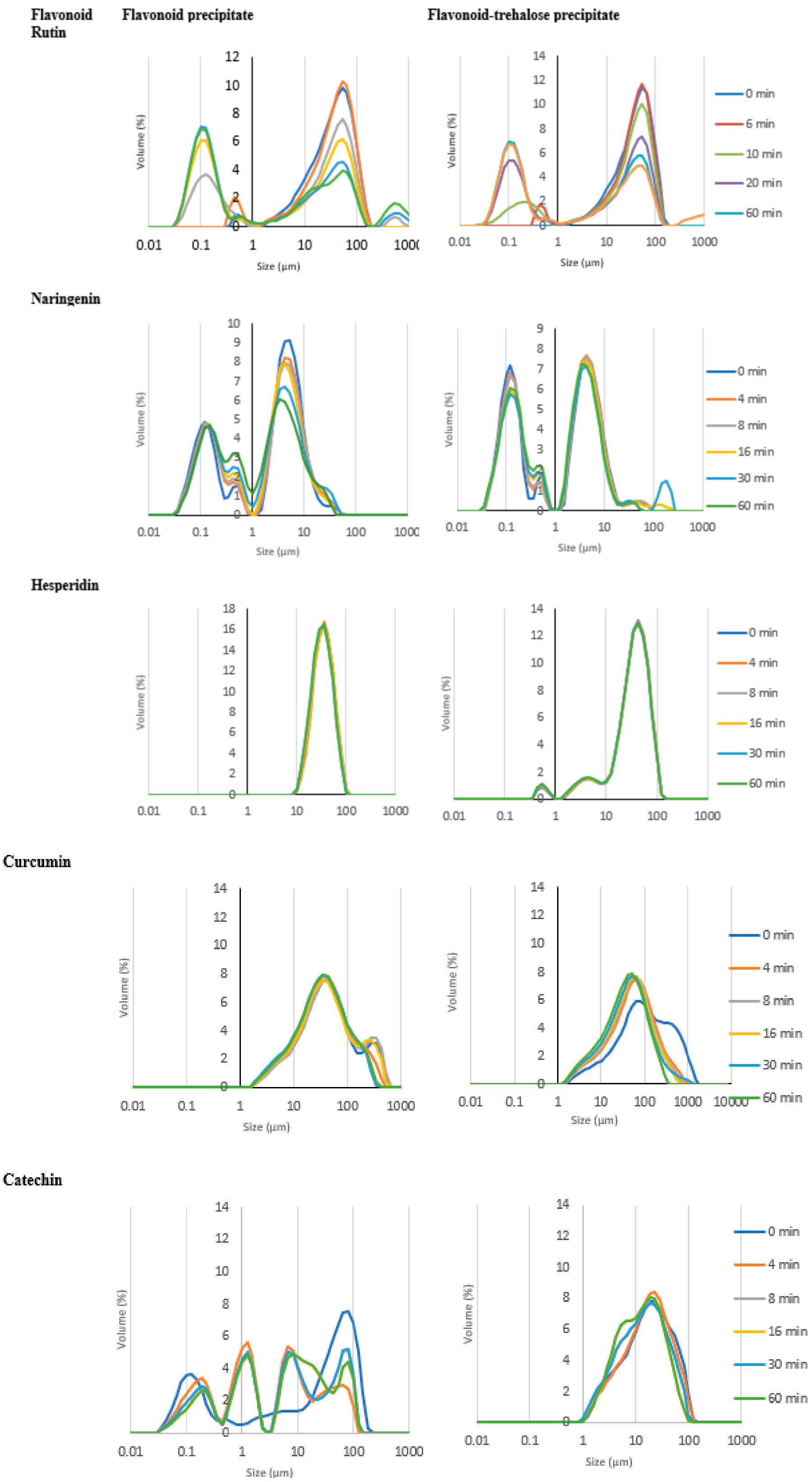

Figure S3. The volume % of particles over time for of the powders of treated polyphenols (precipitates) in the absence and presence of trehalose, dispersed in phosphate buffer (pH 7.0).
